# Mycotic cerebral aneurysm secondary to *Streptococcus parasanguinis* infective endocarditis: a case report

**DOI:** 10.1186/s13256-025-05475-w

**Published:** 2025-08-09

**Authors:** Aakaash Devendra Patel, Christopher Alan Brooks, Peter Gan

**Affiliations:** 1 Department of Critical Care, Waikato Hospital, Pembroke Street, Hamilton, 3204 New Zealand; 2 Auckland, New Zealand; 3 Department of Neurosurgery, Waikato Hospital, Pembroke Street, Hamilton, 3204 New Zealand

**Keywords:** Case report, Infective endocarditis, Mycotic aneurysm, Subarachnoid hemorrhage, Transthoracic echocardiogram, Digital subtraction angiogram

## Abstract

**Background:**

The pathogenesis of infective endocarditis can cause a range of extracardiac complications. Delayed diagnosis may result in catastrophic embolic sequelae. *Streptococcus parasanguinis* is a pathogen that insidiously causes infective endocarditis and has rarely been associated with intracerebral mycotic aneurysms in contemporary medical literature.

**Objective:**

The objective of this cae report is to describe the presentation, investigation, and management of a peculiar case of *S. parasanguinis*-associated infective endocarditis causing a mycotic cerebral aneurysm.

**Case presentation:**

We report our experience in treating a 70-year-old New Zealand European male patient who presented with a left parietal lobe hemorrhage. He was subsequently found to have a mycotic cerebral aneurysm. The patient had underlying *S. parasanguinis* infective endocarditis. This patient was treated neurosurgically for the mycotic aneurysm with subsequent surgical valve replacement. We discuss relevant considerations of treating these pathologies. We discuss the clinical features, cardioradiology and neuroradiology of this obscure but important disease process.

**Conclusion:**

*S. parasanguinis,* a viridans group *Streptococcus*, is an important cause of infective endocarditis but is rarely associated with cerebral mycotic aneurysms. It often causes a subacute form of infective endocarditis, which can hinder initial diagnostic clarity. When embolic phenomena cause the formation of an intracerebral aneurysm, the specific neuroradiological findings of intracerebral mycotic aneurysms should raise the clinician’s suspicion of underlying infective endocarditis. Infective endocarditis becomes significantly more common as people age. Thus, the holistic and objective clinician must maintain a broad differential and investigate widely until a definitive etiology is elucidated. Early recognition is key to favorable outcomes. Management of similar cases requires a multidisciplinary approach with both physicians and surgeons working to identify pathology and provide treatment in a logical sequence.

## Introduction

Cerebrovascular sequelae are known complications of infective endocarditis (IE). The brain and spleen are the most common sites of emboli in left-sided IE, with the overall incidence of cerebral emboli between 10% and 50% and a mortality of more than 30% [1]. The vast majority of these emboli result in cerebral infarction.

Despite a substantial predilection for septic emboli from left-sided IE to deposit within the cerebral vasculature, mycotic aneurysm formation is rare. Mycotic aneurysms account for only 0.7–6.5% of all intracranial aneurysms [2, 9]. The incidence with associated IE is quoted as between 2% and 3% [3, 4].

Mycotic intracerebral aneurysms have a distinct pathophysiology and most commonly occur at terminal arterial branches [5]. This makes them difficult to treat with endovascular techniques hence, open resection is often necessary. Furthermore, in the context of cardiac valvular disease general anesthesia for either intervention carries significant risk.

Concomitant cardiac and neurosurgical pathology poses a considerable challenge for clinicians. Aside from the challenge of elucidating a unifying diagnosis, questions remain surrounding which pathology to target first. In the absence of clear guidelines, this case report aims to add to the existing body of literature. We provide our experience in managing this complex set of pathologies.

We also highlight how the division of medical expertise into specialties, while useful, has its limitations. Human physiology functions as an interdependent set of systems and pathologies are often not limited to one of these systems. We illustrate a rare but pivotal intersection between neurosurgery, cardiothoracic surgery, and cardiology. We aim to demonstrate the necessity of interdisciplinary collaboration to optimize patient outcomes.

## Case presentation

A 70-year-old New Zealand European male patient presented to a peripheral hospital following an episode of dysphasia and out-of-character behavior. He had no notable comorbidities, was taking no regular medications, was an ex-pipe smoker, and was retired and living out of a vehicle modified into a camper van.

Of note, he had been seeing his general practitioner for ongoing nausea, fatigue, and weight loss for several months. Before his presentation with dysphasia, he was admitted under general medicine for cachexia and unintentional weight loss of approximately 20 kg in 2 months. It was noted by the physician caring for him during his initial inpatient admission that he had a mildly raised C-reactive protein. A post-contrast computed tomography (CT) scan of the chest, abdomen, and pelvis identified no evidence of malignancy or embolic phenomena in these regions. Despite this, a referral was made to gastroenterology for endoscopic evaluation of his bowel to rule out gastrointestinal malignancy. Simultaneously, a new audible combined systolic and low-grade early diastolic aortic murmur was also detected. Outpatient transthoracic echocardiogram (TTE) was therefore requested.

**Fig. 1:**
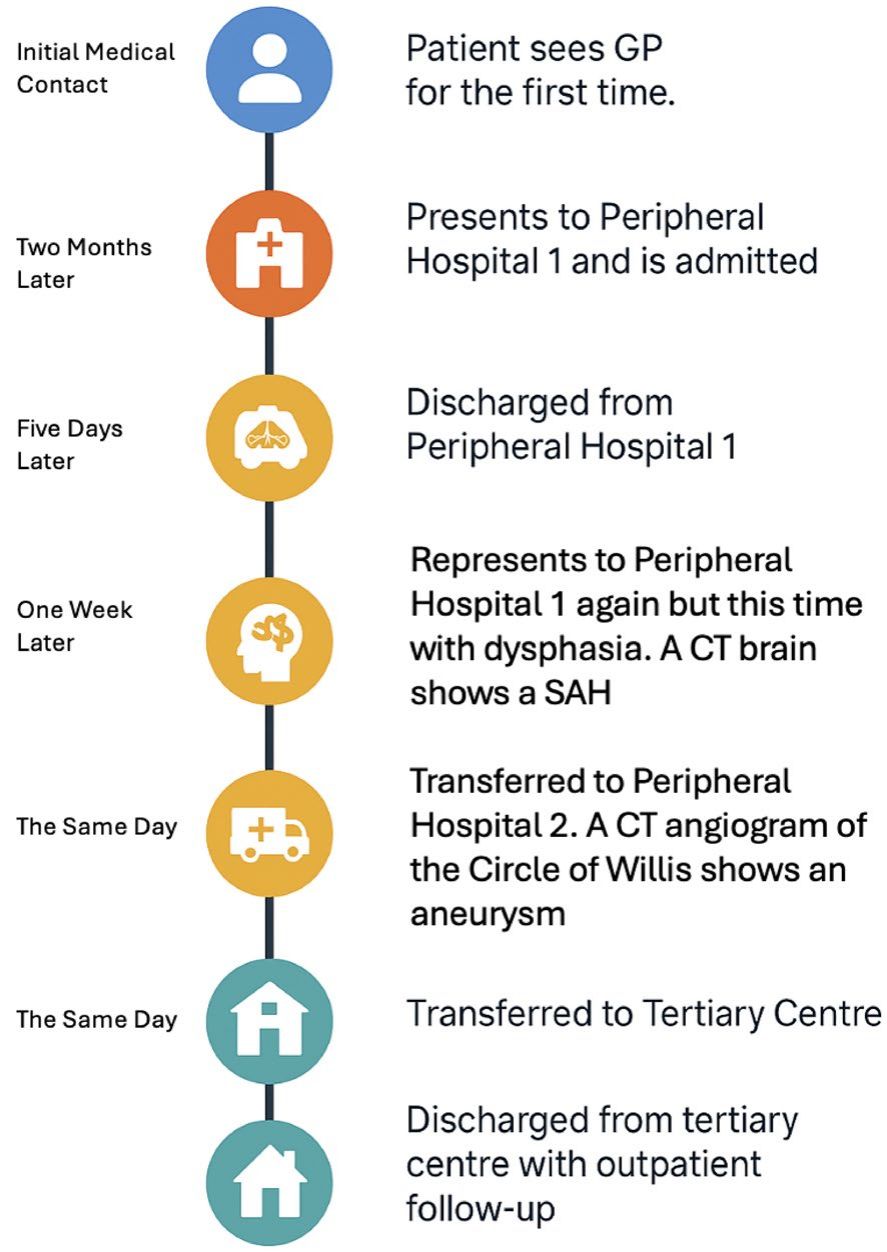
Timeline of events showing patient’s journey from initial medical contact to discharge from our tertiary center

**Fig. 2:**
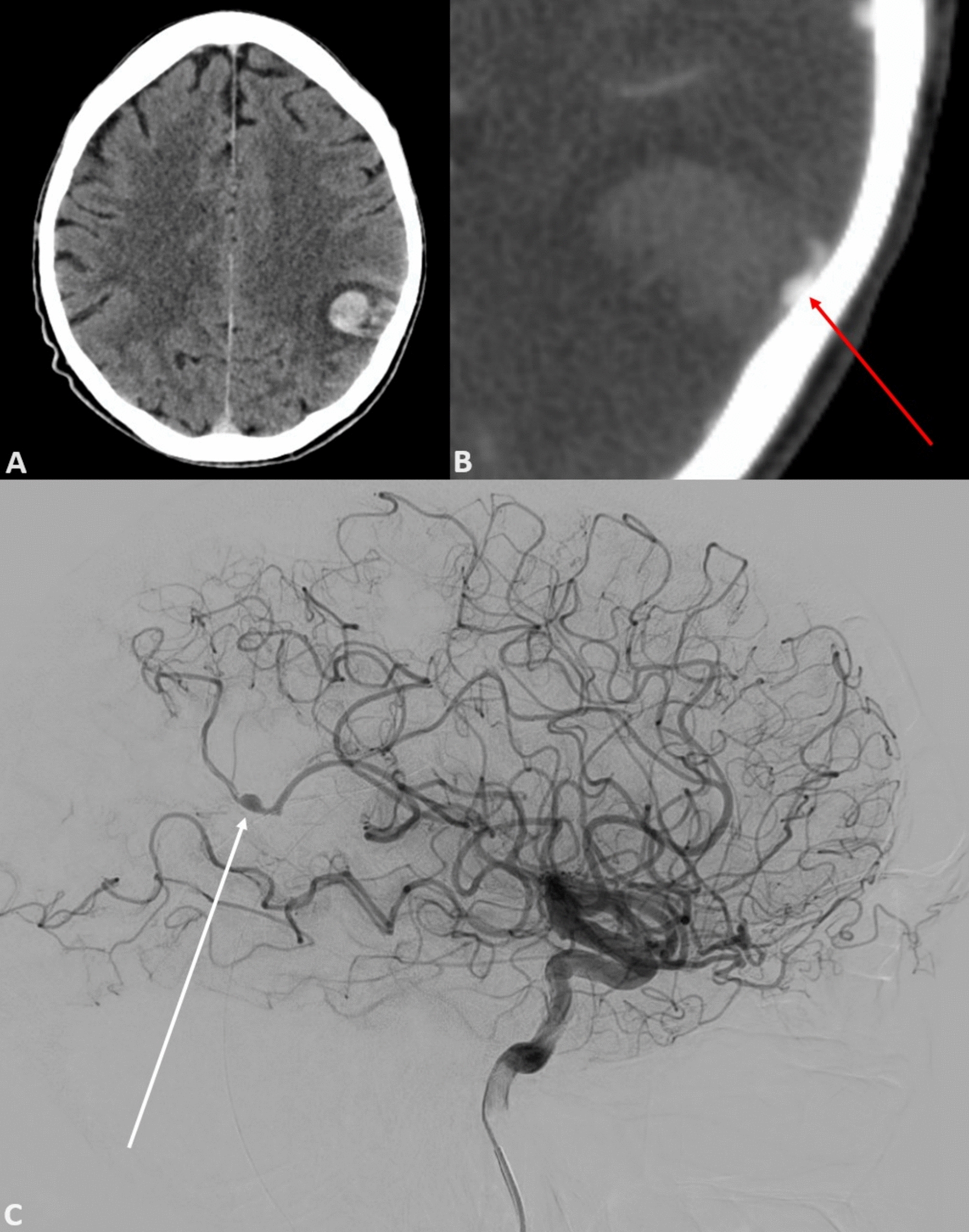
From top right moving clockwise; image **A** showing slice of a non-contrast contrast tomography brain scan showing a left parietal subarachnoid haemorrhage; image **B** showing computed tomography angiogram revealing a left middle cerebral artery branch vessel fusiform aneurysm; image **C** showing still from a digital subtraction angiogram revealing a pseudoaneurysm in relation to the superior parietal branch of the left middle cerebral artery

While awaiting his outpatient investigations, the patient presented to his sister’s residence with confusion and word-finding difficulty. His sister’s concern prompted a visit to a peripheral hospital. Examination revealed the patient had a Glasgow Coma Scale of 14 (E4V4M6) and moderate dysphasia deemed to be predominantly expressive with some receptive component. His right upper limb power was graded as 4/5, while all other limbs had normal power. Right-sided pronator drift was also noted and cranial nerve examination revealed a subtle right-sided facial weakness on smiling. There was no evident sensory deficit. Examination of other systems manifested the aforementioned aortic murmur and significant finger clubbing, but was otherwise unremarkable with no overt signs of cardiac failure or peripheral stigmata of IE. Given the neurological findings, non-contrast CT brain scan was performed, which elucidated a left parietal intracranial hemorrhage (Fig. 2, image A). Patient was then transferred to a larger peripheral hospital with further imaging capabilities. CT angiogram of the Circle of Willis was performed, revealing a left middle cerebral artery (MCA) branch vessel fusiform aneurysm (Fig. 2, image B). Patient was then transferred to our tertiary care center for ongoing management. A timeline of the patient’s journey to our center is outlined in Fig. 1.

At our center, the patient had a digital subtraction angiogram (DSA), which revealed in further detail a pseudoaneurysm of the superior parietal branch of the left MCA (Fig. 2, image C).

It was determined that the patient would need surgical management of his aneurysm. He therefore proceeded to the operating room under neurosurgery for a left parietal stealth-guided craniotomy with clot evacuation and aneurysm securing. The operation was uneventful and the patient had an uncomplicated recovery on the ward.

The patient’s gastroenterologist then contacted the neurosurgical team at our tertiary center expressing concern that the patient may have IE and suggested that blood cultures and a TTE be performed as an inpatient. This raised the suspicion that the patient had suffered a mycotic aneurysm.

**Fig. 3:**
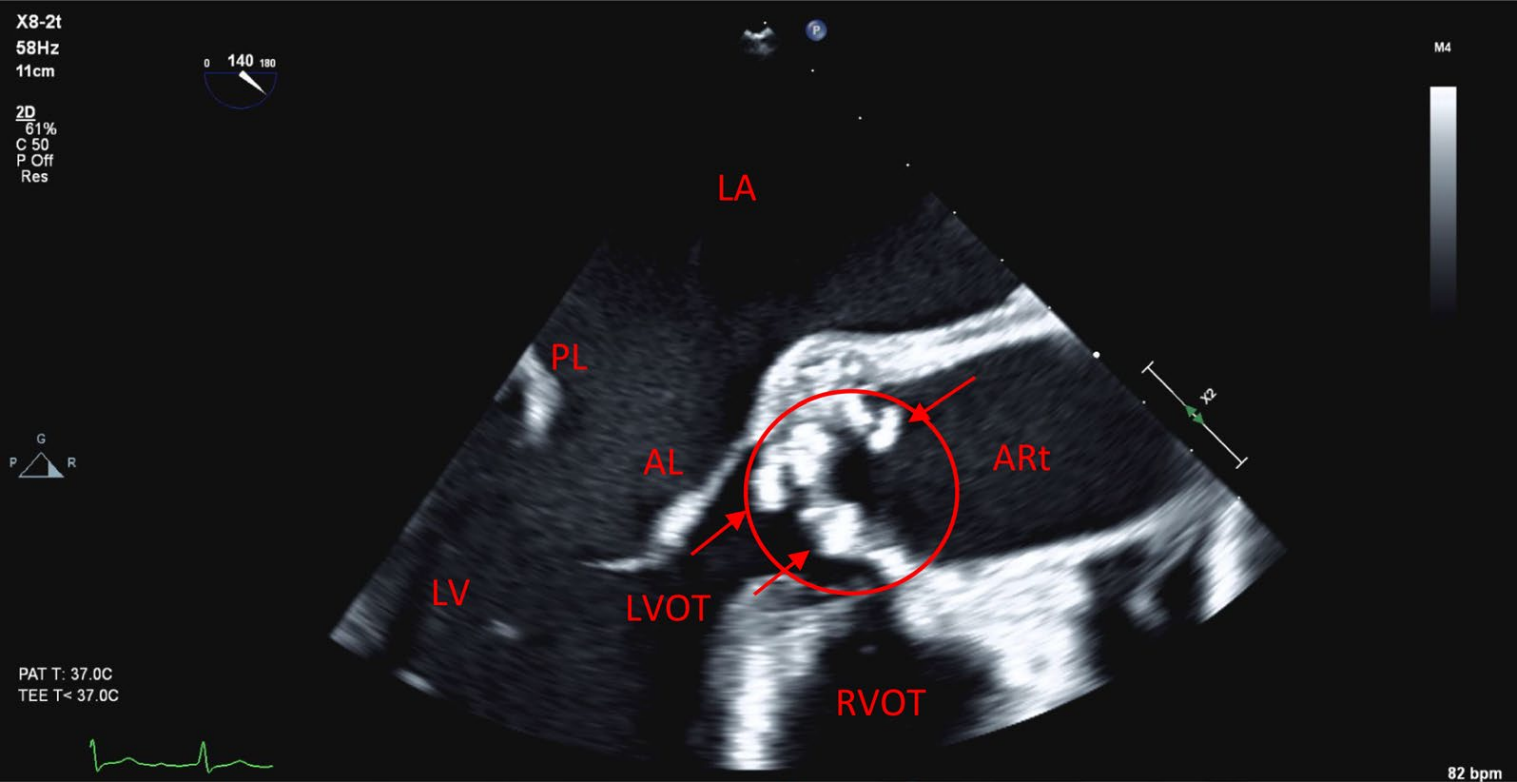
Transesophageal echocardiogram image showing a mid-esophageal long-axis view of the heart with an imaging plane angle of 140 degrees. Pertinent features of this still include the multiple hyperechoic structures on the aortic valve leaflets as indicated by the arrows. On real-time imaging, these were independently mobile of the valve leaflets and were deemed to be infective vegetations. For reference, other structures have been labeled, including left atrium (LA); posterior leaflet of the mitral valve (PL); anterior leaflet of mitral valve (AL); left ventricle (LV); left ventricular outflow tract (LVOT); aortic root (ARt); and right ventricular outflow tract (RVOT). Aortic valve is circled in red

**Fig. 4:**
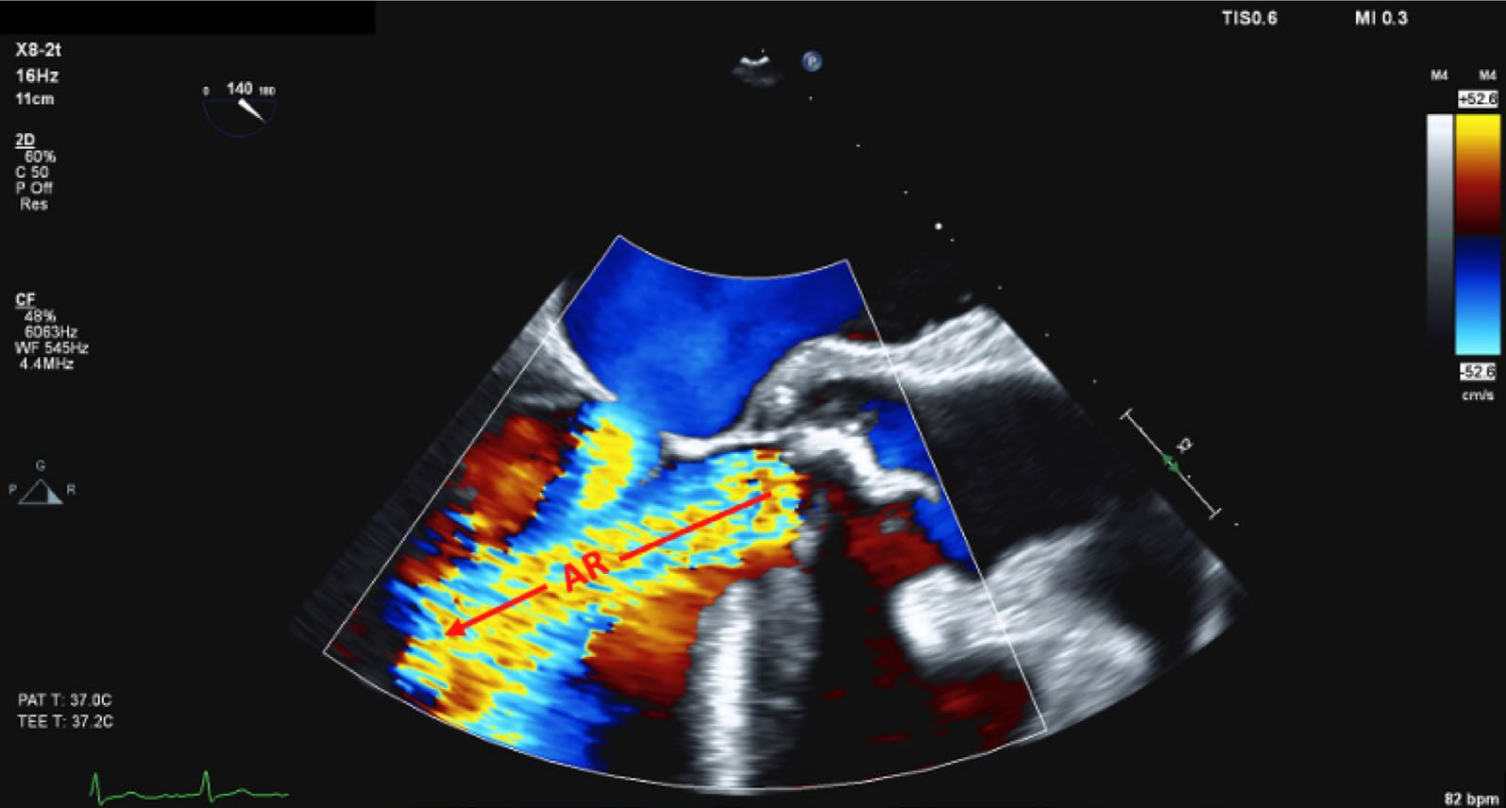
Transesophageal echocardiogram still in mid-esophageal long-axis with an imaging plane of 140 degrees but with color Doppler applied. The labelled structures in image **A** correlate with the above still. As per convention, the color blue denotes flow away from the probe and red towards. Color grading denotes the velocity of the flow in centimeters per second. Jet of aortic regurgitation is labeled “AR,” with an arrow denoting the direction of flow. Other than the jet of aortic regurgitation, it can also be seen that there is antegrade flow across the mitral valve from the left atrium into the left ventricle, denoting that this image was taken during diastole

**Fig. 5:**
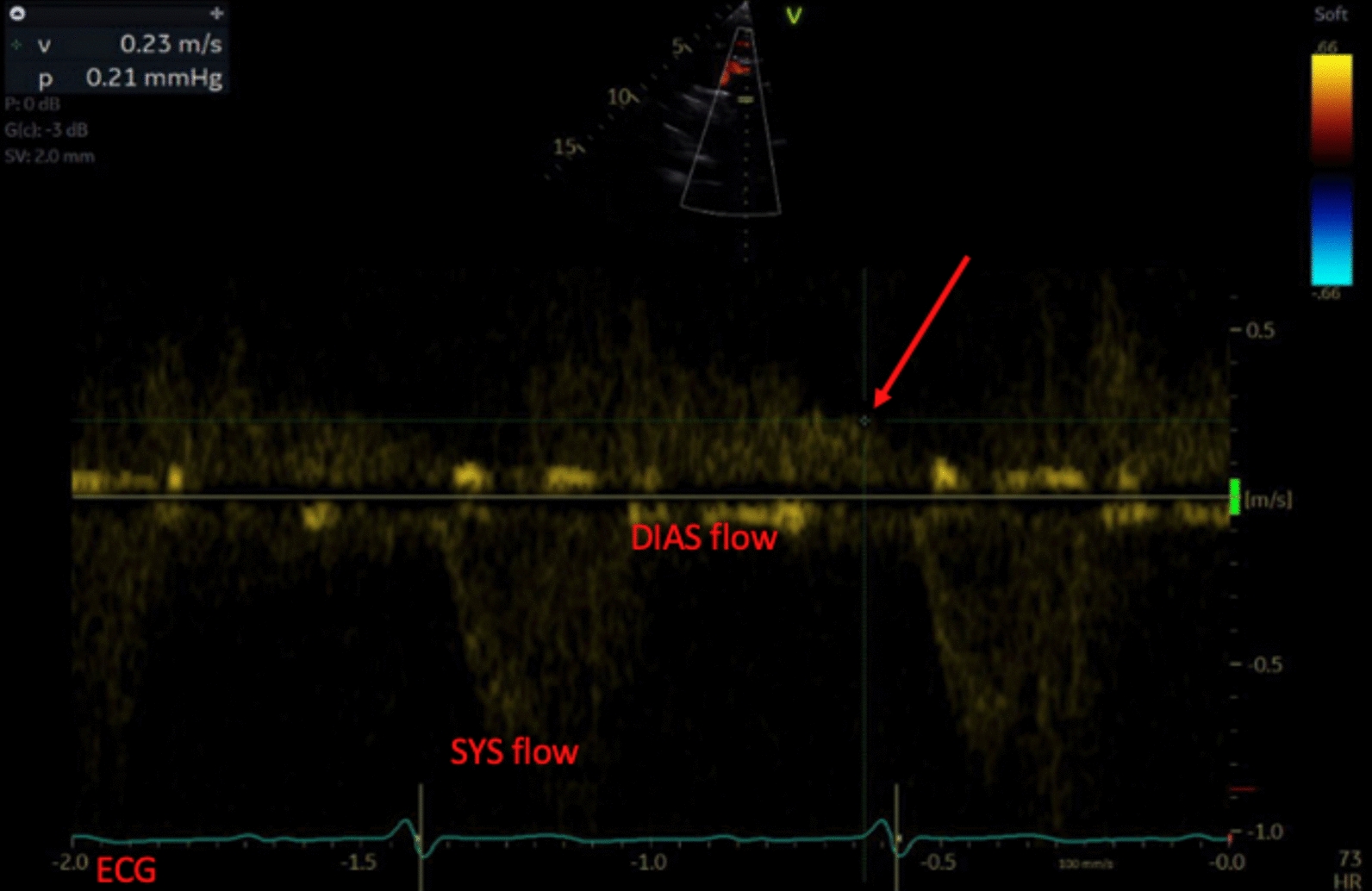
Doppler study of the aortic regurgitation that can be visually demonstrated in image B, although this image was taken using transthoracic echocardiogram. The deflections below the baseline indicate systolic forward flow through the left ventricular outflow tract and past the aortic valve, labeled as “SYS flow.” The scale to the right records this as a positive velocity. The tracing along the bottom of the image denotes a concurrent electrocardiogram performed in real time during the study; it is labeled “ECG.” It can be seen that positive flow through the aortic valve occurs immediately following the QRS complex, indicating that this flow is occurring during systole. Conversely, flow above the baseline is seen between QRS complexes and thus occurs during diastole, this is labeled “DIAS flow.” Deflection above the baseline denotes a negative velocity and indicates retrograde flow across the aortic valve during diastole. It can be seen that retrograde flow does not reach the baseline prior to the commencement of systolic forward flow, this indicates a holodiastolic flow reversal aortic regurgitation. The arrow on the image highlights a velocity measurement taken using Doppler. If this line is followed to the electrocardiogram, it can be seen intersecting it just prior to a QRS complex, denoting that the measurement was taken at the very end of diastole. The velocity at this point in the cardiac cycle is reported in the top right hand corner of the image and is 0.23 m per second, which confirms significant retrograde flow throughout the entirety of diastole

**Fig. 6:**
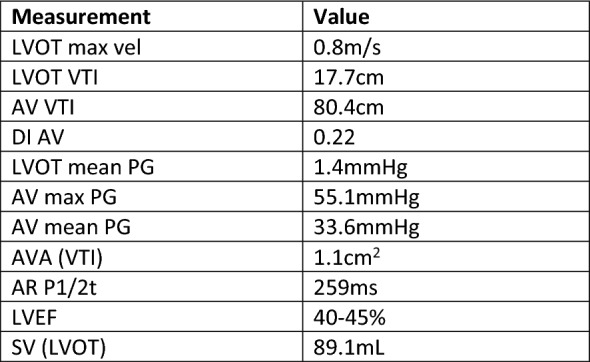
Pertinent quantitative echocardiography metrics as measured on transthoracic echocardiogram. These include the maximum left ventricular outflow tract velocity (LVOT max vel); left ventricular outflow tract velocity time integral (LVOT VTI); aortic valve velocity time integral (AV VTI); dimensionless index of aortic valve (DI AV); left ventricular outflow tract mean pressure gradient (LVOT mean PG); aortic valve maximum pressure gradient (AV max PG); aortic valve mean pressure gradient (AV mean PG); aortic regurgitation pressure half time (AR P1/2t); left ventricular ejection fraction (LVEF); and left ventricular outflow tract stroke volume [SV (LVOT)]

In total, three of six initial blood cultures returned positive for *S. parasanguinis*. The infectious diseases service suggested empiric therapy with benzylpenicillin. The patient had routine blood cultures for 48 hours after the first negative culture returned. None of these returned positive. The patient then proceeded to have TTE, which showed heavily calcified aortic valve leaflets with independently mobile structures. There was severe aortic regurgitation with holodiastolic flow reversal in the proximal descending aorta and early diastolic flow reversal in the abdominal aorta. There was also concomitant moderate aortic stenosis (AS). Normal left and right ventricular size with a mild-to-moderate reduction in left ventricular systolic function was noted. The left ventricular ejection fraction (LVEF) was between 40% and 45%. Pertinent echocardiography images as well as figures are summarized in Figs. 3, 4, 5, 6.

A subsequent transesophageal echocardiogram (TOE) confirmed the diagnosis of aortic valve endocarditis with severe aortic regurgitation (AR) and prolapse of the noncoronary cusp (NCC) into the left ventricular outflow tract (LVOT). The patient was then identified as requiring surgical aortic valve replacement with a bioprosthesis. His surgery was protracted but uneventful. He was routinely admitted to the intensive care unit where he was quickly desedated and extubated. He completed his hospital stay on the cardiothoracic ward and was discharged home without further issue.

The patient completed 4 weeks of intravenous antibiotics. Before discharge, he had a peripherally inserted central catheter placed for completion of his antibiotic course in the community with district nurses. His inflammatory markers were monitored weekly and he was followed up in an infectious diseases clinic prior to cessation of antimicrobial therapy. At follow-up, his white blood cell count and C-reactive protein level had normalized and it was deemed safe to remove his line and discontinue antibiotics. There was no indication for continued treatment with oral antibiotics.

The patient was also followed up routinely by a cardiologist as an outpatient 6 weeks postoperatively. His aortic prosthesis was well seated and functioning normally. His initially reduced ejection fraction (EF) was deemed to be a result of preexisting aortic stenosis. At follow-up, his EF had improved and he had no signs or symptoms of heart failure. He was advised to continue low-dose aspirin for a total of 3 months.

Exactly 8 weeks after discharge, the patient had routine neurosurgical follow-up with repeat brain imaging, including an angiogram. Imaging revealed successful obliteration of the aneurysm with normal vessel patency.

The patient was subsequently discharged back to the care of his general practitioner. At the time of his final follow-up, he was well in the community with no residual neurological deficits. He remained independent and continued to reside alone in his mobile home.

## Discussion

Cerebral manifestations of IE are not uncommon, and clinicians should always maintain a broad differential as to the cause of an embolic event, especially where more than one organ system is involved. In this case, we discussed a mycotic cerebral aneurysm. Because these aneurysms tend to occur at terminal arterial branches [5], the pattern of intracerebral hemorrhage tends to be nonclassical. Typical berry aneurysms generally form where large vessels branch in the Circle of Willis. Rupture therefore causes bleeding in a relatively predictable pattern. Any bleed at a peripheral intracerebral site should be considered suspect for a mycotic aneurysm.

Aneurysm formation begins as bacteria are embedded within the arterial wall. This causes arteritis, which results in a breakdown of the internal elastic lamina and tunica media. The disruption of the arterial wall architecture inhibits the capacity of the blood vessel to absorb pulsatile oscillations in pressure associated with arterial blood flow [5]. As a result, the vessel wall thins and begins to dilate. This leads to a balloon-like or saccular aneurysm forming. Laplace’s law states that the wall tension of a vessel is proportional to its radius; hence, as initial dilatation occurs, the tension on the arterial wall increases, causing further dilatation. This positive feedback loop eventually leads to rupture [9].

IE, independent of aneurysm formation, is considered a life-threatening condition. It involves infection of one or more heart valves. It has an estimated 6-month mortality of 25% [7]. *S. parasanguinis* is a viridans group *Streptococcus*, which is implicated in this case study. While viridans streptococci are commonly implicated in IE, *S. parasanguinis* is rarely the isolated organism in a case where a mycotic aneurysm is a secondary complication. A previously reported case of a 70-year-old female patient with a similar presentation to the one in this case study appears to be the only other documented evidence of this pathology [10].

Patients with IE may present acutely or subacutely depending on the causative organism and host risk factors. Often the presentation can be nonspecific. Similarly, common laboratory investigations such as C-reactive protein and white cell count may be mildly elevated or within the normal range. The clinician needs to retain a high index of suspicion for this pathology to ascertain a timely diagnosis.

The modified Duke criteria is the primary risk stratification tool used to determine clinical risk for IE. It involves collation of both clinical and laboratory information. However, the modified Duke criteria relies on often absent clinical signs [11] and thus cannot be solely relied upon to make a definitive diagnosis.

The mitral valve is the most commonly implicated cardiac structure, followed by the aortic, tricuspid, and pulmonic valves. Importantly, IE can occur without preexisting structural valvular disease.

*S. parasanguinis* is a Gram-positive cocci that belongs to group viridans streptococci. It is an oral commensal organism that expresses a number of adhesins and collagen-binding proteins (CBPs) that allow it to adhere to tissues of the host. *S. parasanguinis* likely uses many of these adhesins and CBPs to bind with connective tissues deep to damaged endocardium and evade innate immune destruction [6].

Unlike *Staphylococcus* spp., streptococci cannot directly penetrate the endocardial layer of native tissue valves. *Streptococcus* spp. generally require the presence of nonbacterial thromboemboli (NBTE) on heart valves to adhere and form infective vegetations. NBTEs are aggregates of platelets covered with a fibrin mesh. They appear when the exposed collagen elements forming the subendocardial valvular architecture are unconcealed to circulating platelets. The endocardium ordinarily overlies connective tissue that, when exposed to blood flow, is procoagulable [8, 12].

The inability of *Streptococcus* spp. to directly adhere to undamaged valvular tissue gives this phenotype of IE a much more indolent natural history when compared with its staphylococcal counterpart. This subacute pathogenesis means that presentation to a healthcare provider may occur primarily as a result of an embolic complication of IE.

In cases in which a patient presents with a neurological deficit and subarachnoid hemorrhage (SAH) is identified, cerebral angiography is mandatory. The advent of multidetector CT (MDCT) imaging has significantly improved image resolution and the capacity for noninvasive imaging [13]. However, DSA remains the gold standard for assessing cerebral vessels, with a reported sensitivity of 99% [14].

DSA is generally required as part of diagnostic workup [14] and remains the standard of care at our institution. While CT angiography may assist with detection of larger classical aneurysms, it can fail to identify the cause of SAH in 5–30% of cases [14]. Small mycotic aneurysms that occur at distal branches may be missed on conventional CT-based imaging. While CT angiography did reveal an aneurysm in our patient, it was not deemed to be of sufficient resolution to fully characterize its location, morphology, and size. Furthermore, the subsequent DSA we performed allowed assessment of the remaining cerebral vasculature in greater detail. This gave us confidence that smaller vascular malformations, which could be missed on CT, were not present. It also confirmed that a neurointerventional approach to treatment was not feasible; a fact not apparent from assessment of CT images alone. This case demonstrates the importance of identifying the cause of SAH and not simply treating it as an isolated entity.

Another pertinent discussion point surrounding the cerebral pathology in this case is the choice of treatment for the aneurysm. Previous small-scale studies have shown that overall mortality is significantly lower with intervention compared with antibiotic therapy alone. One study cites a 13% mortality with intervention and 40% with isolated antimicrobial treatment [15]. Therapeutic options for intracerebral aneurysms include either endovascular procedures (coiling or flow diverting stent) or craniotomy with open securing. Most often, patients with mycotic aneurysms are committed to a craniotomy given that the malformations tend to be too distal for catheter-based approaches. However, where possible, endovascular treatment is preferred in certain patient cohorts. Where acute cardiac valve replacement is required before neurointervention, and a mechanical prosthesis is used, endovascular treatment is preferable if possible. This prevents the need for anticoagulant reversal prior to craniotomy [15].

The patient discussed in our case was initially investigated for the possibility of malignancy. While a differential diagnosis of malignancy in a patient of this age with chronically raised inflammatory markers and weight loss is reasonable, it is crucial to avoid developing a diagnostic bias for malignancy in older patient cohorts. It is important to recognize that these findings, while commonly associated with malignancy, are nonspecific. Up to 90% of patients with IE present with systemic symptoms similar to those seen in cancer, such as weight loss, malaise, anorexia, fevers, and chills [16]. A multifaceted approach to the investigation of systemic symptoms should be utilized until a clear cause is elucidated. In Western countries, up to one-third of IE cases are in patients older than 70. The elderly are also estimated to have a fivefold increase in risk for developing IE [16]. Furthermore, the consequences of undiagnosed IE in this patient cohort are significant. IE is a curable condition; however, owing to reduced physiologic reserve, elderly patients who are diagnosed late may never return to their premorbid state.

## Conclusion

We have presented a case of a 70-year-old male patient who was found to have a mycotic pseudoaneurysm of the superior parietal branch of the left MCA secondary to indolent *S. parasanguinis* IE of the aortic valve. He was treated with antibiotics, craniotomy, and securing of the aneurysm; followed by bioprosthetic aortic valve replacement.

This case exemplifies how streptococcal IE can be an indolent disease with features that can mimic malignancy. Maintaining a broad differential and investigating multiple possible etiologies in patients who present with nonspecific constitutional signs is advisable.

TTE is a safe, affordable, and increasingly accessible cardiac imaging modality. It should form part of investigation whenever older patients present with nonspecific constitutional symptoms. It should be especially employed when atypical cerebral aneurysms are seen, even if blood work is relatively unremarkable.

While CT imaging is becoming increasingly accurate, DSA remains the standard of care for neurovascular imaging. CT angiography can be falsely reassuring when mycotic aneurysms are suspected. This is because these aneurysms are often small, of atypical morphology, and located in distal vascular branches. They can therefore frequently be mistaken for artifact.

When neuroimaging reveals atypical hemorrhage sites or aneurysms at nonbranch points in the cerebral vasculature, clinicians should suspect infectious causes.

There appear to be few cases of mycotic cerebral aneurysms resulting specifically from IE attributable to *S. parasanguinis*. This case study aims to supplement the medical literature and provide an anecdotal framework of how to investigate and treat such a patient.

## Data Availability

The data presented in this article concerns an individual patient and its source is not publicly available for privacy reasons.

## Code availability

Not applicable to this study.
